# A longitudinal study of the antilipolytic effect of insulin in women following bariatric surgery

**DOI:** 10.1038/s41366-021-00914-2

**Published:** 2021-07-28

**Authors:** Kelvin Ho Man Kwok, Daniel P. Andersson, Mikael Rydén, Peter Arner

**Affiliations:** 1 Department of Biosciences and Nutrition, Karolinska Institutet, Stockholm, Sweden; 2 Department of Medicine (H7), Karolinska Institutet at Karolinska University Hospital, Stockholm, Sweden

**Keywords:** Obesity, Fatty acids

## Abstract

Insulin resistance of glucose utilization is fully restored following BMI normalization after bariatric surgery. We investigated if this also pertains to insulin-induced effects on fatty acid handling. Forty-three women with obesity (OB) were investigated before and 2 years after Roux-en-Y gastric by-pass when BMI was <30 kg/m^2^ (PO) and compared with 26 never obese women (NO). The Adipo-IR index was used as measure of insulin antilipolytic sensitivity. Changes (delta) in circulating glycerol and fatty acid levels during hyperinsulinemic euglycemic clamp represented the insulin maximum antilipolytic effect. Overall fatty acid utilization was reflected by delta fatty acids minus 3 × delta glycerol. Adipo-IR was higher in OB than in NO and PO (*p* < 0.0001), the latter two groups having similar values. Insulin lowered glycerol levels by about 70% in all groups, but delta glycerol was 30% larger in PO than in NO (*p* = 0.04). Delta adds and adds utilization were similar in all groups. We conclude that women with obesity, whose BMI is normalized after bariatric surgery, have improved maximum in vivo antilipolytic effect of insulin above expected in absolute but not relative terms as regards glycerol changes, while the handling of circulating fatty acids is changed to the normal state.

## Introduction

Insulin inhibits lipolysis in fat cells through spare receptors [1]. Therefore, adipose insulin resistance at early signal steps is mirrored by the half maximum effective concentration (insulin sensitivity), while resistance at distal steps at very high hormone concentrations reflects maximum action (responsiveness). Subjects with obesity or overweight display decreased sensitivity but normal responsiveness for the antilipolytic effect in fat cells [2]. Insulin resistance may also involve additional fatty acid metabolic events [3].

In post-obesity, in vivo insulin-induced glucose metabolism is completely normalized [4]. Whether the same pertains to antilipolysis and/or fatty acid utilization is unknown although reduction of BMI from about 43 to 32 kg/m^2^ normalized the antilipolytic effect of insulin in vitro [5].

We presently investigated the effects of insulin on circulating fatty acids in vivo. Women with obesity were investigated before (OB) and 2 years after gastric by-pass when they had attained a post-obese state (PO). They were compared with never obese women (NO). Changes in circulating fatty acids and glycerol during hyperinsulinemic euglycemic clamp measured insulin responsiveness. Antilipolytic insulin sensitivity was estimated by the so-called Adipo-IR index [6], which correlates strongly with antilipolytic sensitivity in vivo and in vitro [7, 8].

## Material and methods

The patients were from a longitudinal study (NCT01727245) of Roux-en-Y by-pass (RYGB) surgery for obesity. This sub-study examined 43 women before and 2 years after RYGB when they reached a PO state (BMI < 30 kg/m^2^). For comparison, 26 never obese healthy control women (NO) were recruited. None had severe chronic disorder, diabetes, or regularly used pain killers. Twelve had hypertension which remained in five patients at follow-up. Postoperative dietary instructions were given as described [4]. The study was approved by the regional ethics committee in Stockholm and informed written consent was obtained.

Examinations were performed in the morning after an overnight fast. The menstrual cycle was not considered as it does not seem to influence lipolysis or adipocyte insulin action. Venous blood was used for routine clinical chemistry measures. Adipo-IR was calculated from serum insulin and fatty acid values [8]. There is no consensus on how to measure the antilipolytic action of insulin in vivo [6]. We performed a 2-h standard high-dose insulin clamp investigation for responsiveness at 10 a.m. as described [4]. Glucose disposal rate was determined during the last hour (*M*-value). The insulin effect was calculated as differences (delta) in serum glycerol or fatty acids values between the end (115 min) and start (−5 min) of clamp. We also calculated % inhibition of glycerol levels. The overall fatty acid utilization was reflected as delta (µmol/l) fatty acids minus 3 × delta glycerol. The breakdown of one triglyceride molecule during lipolysis generates one glycerol and three fatty acids. The validity of the calculation rests on several assumptions. Glycerol is not significantly metabolized by fat cells but mainly by the liver. In the fasting state, glycerol can to some extent be produced by hydrolysis of circulating triglycerides [9]. There is no report that insulin influences liver glycerol metabolism such as gluconeogenesis or triglyceride assembly or hydrolysis of circulating triglycerides. Therefore, delta glycerol mainly reflects lipolysis changes. Fatty acids are produced and re-esterified by fat cells, oxidized mainly in skeletal muscle, used by the liver in the production of triglycerides in very low-density lipoproteins (VLDL-TG). They are also trapped within the adipose tissue extracellular space. Oxidation and VLDL-TG production are inhibited in vivo by insulin [10, 11]. Re-esterification and fatty acid trapping are stimulated by insulin [12, 13]. Our protocol does not distinguish between these forms of fatty acid utilization. We assumed that the distribution volumes for glycerol and fatty acids were the same in all groups and that elimination rates of glycerol and fatty acids were in steady state during the last hour of clamp as for glucose disposal rate.

Glycerol was determined by a specific bioluminescence method [14] and insulin by ELISA (Mercodia, Sweden). Fatty acids were measured using a specific colorimetric kit (FUJIFILM Wako Chemicals Europe GmbH, Germany). Some fatty acid values were below detection limit and treated as zero.

Values are mean ± SD in text and tables and as described in legend to figures. Delta glycerol and fatty acids were primary outcome variables. NO was compared with OB and PO by unpaired *t*-test. OB and PO were compared by paired *t*-test and analysis of covariance (ANCOVA). We compared changes in antilipolysis with changes in clinical variables after RYGB using linear regression. All statistical tests were two-tailed with *p* < 0.05 as statistically significant.

## Results

Clinical data are in Table 1. Body weight decreased by 33 ± 7% following RYGB (*p* < 0.0001). OB displayed expected clinical abnormalities which normalized after RYGB to values comparable with NO, except for slightly lower insulin in PO. Adipo-IR values were increased in OB and decreased to NO levels in PO.

**Table 1:** Clinical characteristics in women.

				*p* value		
	NO (*n* = 26)	OB (*n* = 43)	PO (*n* = 43)	NO vs. OB	OB vs. PO	NO vs. PO
Age (years)	44 ± 12	43 ± 10	45 ± 10	0.64	<0.0001	0.74
Body weight, kg	69 ± 8	106 ± 12	71 ± 8	<0.0001	<0.001	0.40
BMI (kg/m^2^)	24.4 ± 2.6	38.4 ± 3.0	25.6 ± 2.7	<0.0001	<0.0001	0.12
Waist circumference (cm)	88 ± 10	120 ± 7	90 ± 7	<0.0001	<0.0001	0.28
Waist-to-hip ratio	0.87 ± 0.07	0.98 ± 0.07	0.90 ± 0.06	<0.0001	<0.0001	0.19
fS-insulin mU/l	6.1 ± 3.1	12.6 ± 6.2	4.6 ± 1.6	<0.0001	<0.0001	0.0071
*M*-value mg/kg/min	12.1 ± 2.6	9.9 ± 3.4	11.7 ± 2.5	0.004	<0.0001	0.57
fP C-reactive protein, mg/l	1.9 ± 3.2	6.0 ± 7.1	0.7 ± 1.0	0.0007	<0.0001	0.30
fP-glucose (mmol/l)	5.1 ± 0.4	5.4 ± 1.0	5.2 ± 0.5	0.13	0.12	0.33
fP-triglycerides (mmol/l)	0.89 ± 0.39	1.33 ± 0.53	0.81 ± 0.32	0.0004	<0.0001	0.40
Adipo-IR, units	3.7 ± 2.6	9.1 ± 4.8	2.9 ± 1.9	<0.0001	<0.0001	0.35

Values are given as mean ± SD. They were compared by paired (OB vs. PO) or unpaired (NO vs. OB and PO) *t*-test. *M*-value was related to lean body mass.

*NO* never obese, *OB* obese, *PO* post-obese, *fS* fasting serum, *fP* fasting plasma.

There was a rapid steady-state elevation of insulin values during the clamp (Fig. S1A) and a swift decrease in glycerol levels, which leveled off at about one-third of the initial values (Fig. S1B). Thus, insulin was not able to clear the circulation of glycerol. In contrast, circulating fatty acid levels decreased to almost zero in all three conditions (Fig. S1C).

**Fig. 1:**
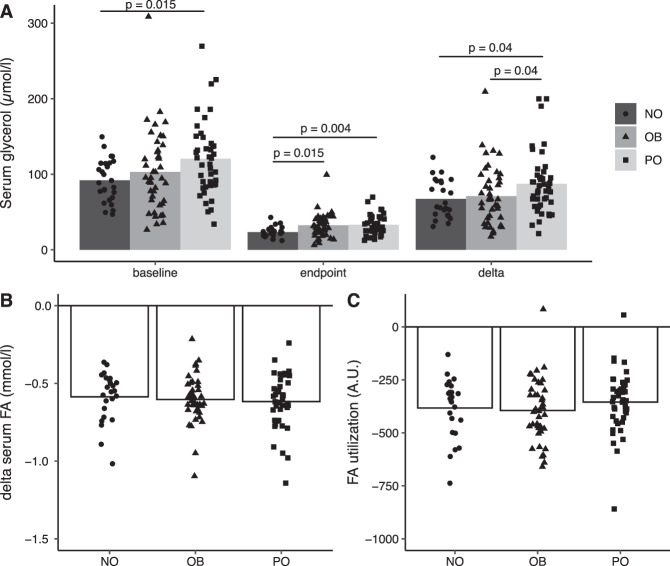
Findings on serum glycerol and fatty acids (FA) during clamp. **A** is absolute glycerol levels at −5 min (baseline) and 115 min (endpoint) of clamp as well as delta values (levels at −5 min subtracted by the value at 115 min). **B** is delta for fatty acid levels (115-min values subtracted from −5 min value, **C** is delta for fatty acid re-utilization calculated as in **B**. Values are mean and individual. Women before (OB, obese state) and after (PO, post-obese state) bariatric surgery were compared by paired *t*-test. Never obese women (NO) were compared with OB and PO by unpaired *t*-test. Only significant results are shown (*p* < 0.05). The mean ± SD values for delta glycerol (µmoles/l) were 67 ± 25, 71 ± 40, and 87 ± 41 in NO, OB, and PO, respectively.

Absolute values for glycerol are recorded in Fig. 1A. At −5 min, they were similar in OB and NO but higher in PO than NO (*p* = 0.015). At 115 min of clamp, OB and PO had similar values but higher than in NO (*p* ≤ 0.015). Changes in glycerol (delta) were calculated. Delta glycerol was comparable in NO and OB but 30% greater in PO than in NO (*p* = 0.04). The difference remained after correction for −5 min glycerol value by ANCOVA (*F* = 4.1; *p* = 0.048). However, in relative terms inhibition of glycerol levels by insulin was similar in NO, OB, and PO (73 ± 8%, 67 ± 8%, and 71 ± 7%, respectively). For delta fatty acids (Fig. 1B) or fatty acid utilization (Fig. 1C), no significant differences between groups were recorded. Antihypertension therapy did not influence the results. No relationship was observed between changes in C-reactive protein, *M*-value, or % body weight decrease and delta glycerol following RYGB.

## Discussion

Adipose insulin resistance may develop before other metabolic alterations and directly contribute to impaired insulin action in skeletal muscle and liver [15]. It is apparent that obesity has different impact on glycerol and fatty acid handling by insulin.

We studied insulin action indirectly. Changes in glycerol and fatty acid levels represented maximum insulin action and Adipo-IR reflected insulin sensitivity. Therefore, the interpretation of the data rests on several assumptions regarding appearance and removal of glycerol and fatty acids from the circulation. Insulin has additional effects on fatty acid handling besides antilipolysis as thoroughly discussed in “Material and methods”. For technical reasons it was not possible to directly examine these events (oxidation, VLDL-TG production, adipose trapping). Insulin may also have hitherto unrecognized effects on glycerol metabolism. Therefore, it is not known if some of these actions of insulin are differentially altered after RYGB treatment. However, by comparing changes in fatty acids and glycerol during the clamp we could get a reflection of total fatty acid utilization, which was not altered in obesity or changed after RYGB treatment. Furthermore, insulin almost fully suppressed circulating fatty acid levels in all conditions, suggesting that the maximum action of insulin on fatty acid handling is not influenced by obesity. On the other hand, the sensitivity of antilipolysis and thereby insulin sensitivity of fatty acid handling (Adipo-IR) is clearly reduced in obesity but fully normalized in the post-obese state. In absolute terms the maximum antilipolytic effect (delta glycerol) is not altered by obesity but increased by 30% in the post-obese state compared with the never obese state. However, in relative terms this maximum effect was similar in all three conditions (about 70% inhibition).

Our results relate to lipolysis in the morning after an overnight fast when both Adipo-IR (at 8 a.m.) and clamp (at 10 a.m.) were performed. Diurnal measures of lipolysis show that the rate is highest by then and not related to insulin status [16]. We only examined women and sex influences lipid metabolism [17, 18]. Therefore, we cannot extrapolate our findings to men. We also do not know the molecular mechanism(s) behind the findings. It is unlikely that the observed effects are explained by the surgical treatment as bariatric surgery and conservative weight-reducing therapy have similar effects on metabolic outcome [19].

Altogether, we propose the following model for insulin action in vivo on glycerol and fatty acids in obesity. In OB, antilipolytic sensitivity, but not effect responsiveness, is decreased implying that fatty acid levels can be almost completely suppressed by high levels of insulin. In PO the sensitivity of antilipolysis is normalized. The absolute maximum effect is increased above the normal level and might be of importance for lower energy expenditure and lipid oxidation rates reported in formerly obese persons [20]. However, the relative maximum antilipolytic effect is not influenced by obesity or weigh reduction (70% decrease of starting glycerol level). The maximum effect of insulin on fatty acid handling appears not to be influenced by body weight status.

In summary, long-term body weight reduction to a non-obese state leads to enhanced antilipolytic effect of insulin in absolute but not relative terms when glycerol levels are investigated. Nevertheless fatty acid utilization is normal.

## Supplementary information

Figure S1
