# Supplementary material for: A longitudinal study of the antilipolytic effect of insulin in women following bariatric surgery
Source: Int J Obes (Lond). 2021 Jul 28;45(12):2675–8. doi: 10.1038/s41366-021-00914-2 (PMC8606310; doi:10.1038/s41366-021-00914-2)
Supplement: Supplementary file 1 — Figure S1 [file 41366_2021_914_MOESM1_ESM.pdf]

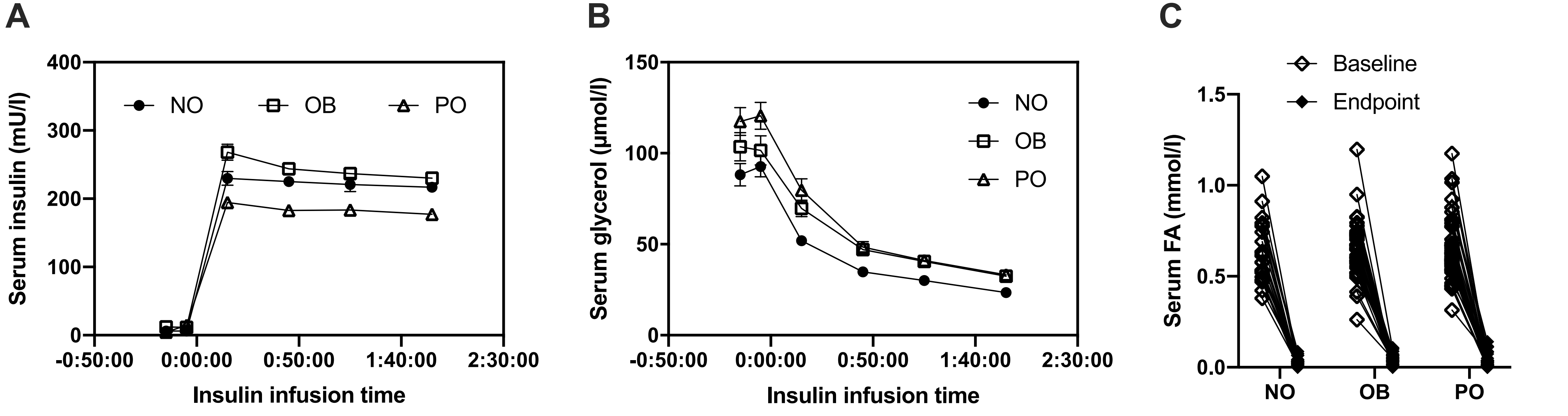
A
400
300
200
100
0
Serum insulin (mU/l)
NO
OB
PO
-0:50:00
0:00:00
0:50:00
1:40:00
2:30:00
Insulin infusion time
B
150
100
50
0
Serum glycerol (µmol/l)
NO
OB
PO
-0:50:00
0:00:00
0:50:00
1:40:00
2:30:00
Insulin infusion time
C
Baseline
Endpoint
1.5
1.0
0.5
0.0
Serum FA (mmol/l)
NO
OB
PO

Figure S1. Findings on serum insulin, glycerol and fatty acid. **A** and **B** are insulin and glycerol levels before and during the hyperinsulinemic euglycemic clamp investigation. **C** is fatty acids before (baseline) and at the end of clamp. **A** and **B** show mean ± standard error of mean. **C** shows individual values. NO=never-obese control women. OB=women with obesity before Roux-en-Y gastric by-pass (RYGB). PO=women after RYGB when they reached a post-obese state.
